# Prediction of improvement after anterior cruciate ligament reconstruction

**DOI:** 10.1515/med-2021-0300

**Published:** 2021-05-29

**Authors:** Mikołaj Wypych, Robert Lundqvist, Dariusz Witoński, Rafał Kęska, Anna Szmigielska, Przemysław T. Paradowski

**Affiliations:** Department of Emergency Medicine, Stanisław Rybicki Regional Hospital, Skierniewice, Poland; Research and Innovation Unit, Norrbotten County Council, Luleå, Sweden; Chair of Clinical Physiotherapy, Institute of Health Sciences, Social Academy of Sciences, Łódź, Poland; Department of Orthopedics, Poddębice Health Centre, Poddębice, Poland; Department of Cardiology, Władysław Biegański Medical University Hospital, Łódź, Poland; Department of Surgical and Perioperative Sciences, Division of Orthopedics, Sunderby Research Unit, Umeå University, Sunderby Central Hospital of Norrbotten, SE-971 80 Luleå, Sweden; Faculty of Health Sciences, Ludwik Rydygier Collegium Medicum, Nicolaus Copernicus University in Toruń, Jagiellońska 13/15, PL-85-067 Bydgoszcz, Poland; Department of Clinical Sciences Lund, Clinical Epidemiology Unit, Orthopedics, Lund University, SE-221 85 Lund, Sweden

**Keywords:** anterior cruciate ligament reconstruction, KOOS, outcome improvement, decision making, knee function

## Abstract

**Objective:**

The retrospective investigation was carried out to assess whether subjects who fulfilled our proposed recruitment criteria responded more favorably to anterior cruciate ligament reconstruction (ACLR) than those who did not.

**Methods:**

We retrospectively analyzed 109 skeletally mature subjects (78 men and 31 women) according to the following proposed criteria of recruitment: (1) pre-injury Tegner activity score ≥7 and a wish to return to a professional sports activity, (2) residual knee instability following injury and/or (3) age <20 years at the operation. The primary outcome was an improvement between assessment A (before operation) and B (mean follow-up of 1.6 years) in the average score for four of the five Knee injury and Osteoarthritis Outcome Score (KOOS) subscales, covering pain, symptoms, difficulty in sports and recreational activities, and quality of life (KOOS_4_).

**Results:**

The proposed recruitment criteria for ACLR were met by 58 subjects (53%). There were 49 subjects (45%) who improved between assessment A and B. Subjects who met proposed recruitment criteria were more likely to improve clinically after ACLR (OR 5.7, 95% CI 2.5–13.3).

**Conclusions:**

Fulfillment of proposed recruitment criteria was a strong predictive factor for outcome improvement in short- to medium-term follow-up after ACLR.

**Level of evidence:**

Case-control study. Level of evidence 3.

## 1 Introduction

Reconstruction of the torn anterior cruciate ligament (ACL) is one of the most frequently performed operations in orthopaedic surgery [1]. The rationale for the operation is primarily to improve knee function and stability and secondly to prevent additional injury to the knee and thus reduce the risk of post-traumatic osteoarthritis [2].

ACLR is perceived to be an effective operation in young and active subjects; however, clinical outcomes are often unsuccessful [3,4,5].

A return to earlier activity can be affected by both decreased knee function and a fear of further knee injury or reinjury [6]. A recent meta-analysis showed that 83% of elite [7] and only 60% of non-elite athletes [8] returned to their pre-injury activity level. A large study of 1,761 young individuals found that over one in three reported knee difficulties 6 years following primary ACLR [9]. The rate of those practising sport at pre-injury or higher level was reported to be about 40% after 2 years [10] and only 20% after 5 years [11] after ACLR. It has already been confirmed that ACL rupture does not automatically cause functional impairment and instability and that many ACL-deficient individuals have the potential to return to their pre-injury activities for a limited period without ACLR [5].

We hypothesize that poor outcomes of ACLR may reflect inaccurate selection of candidates for operation. Currently, there is no consensus regarding ACLR recruitment criteria, and the surgeon’s decision to qualify for the operation is more authority-based rather than evidence-based. In order to find the subjects who are less likely to improve following the surgery, determinants contributing to subjectively perceived successful outcomes should be identified. Thus, the purpose of the study was to investigate retrospectively whether subjects who would have met our proposed criteria, actually responded better to ACLR.

## 2 Materials and methods

### 2.1 Subjects

A prospective study was conducted on subjects who had undergone ACLR at the Department of Reconstructive Surgery and Arthroscopy of the Knee Joint, Medical University in Łódź, Poland, between January 2007 and November 2012. Subjects’ eligibility for ACLR was assessed by an orthopedic surgeon who later performed the operations (DW).

**Figure 1:**
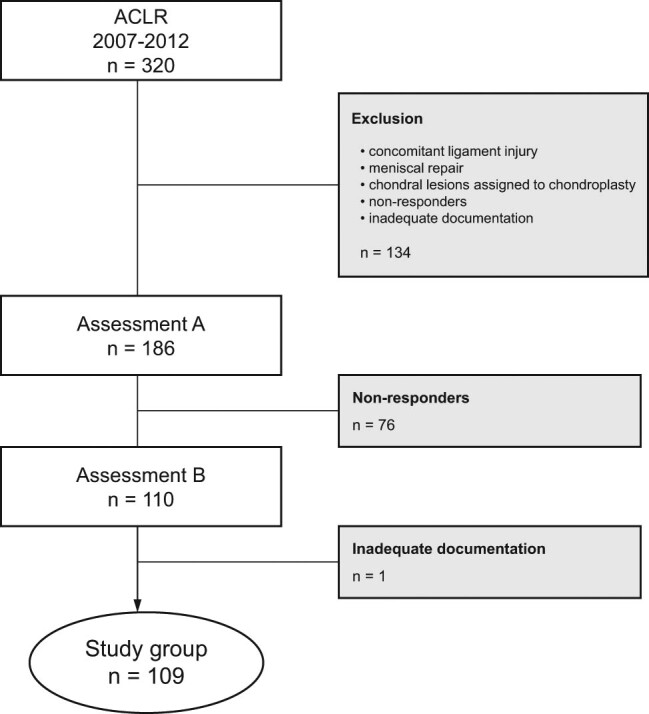
Flowchart presenting the study group formation.

Inclusion criteria were skeletal maturity, signs of ACL tear based on clinical examination and MRI (if considered necessary), subject’s request to maintain an active lifestyle and ability to speak Polish. Patients were excluded if they had additional collateral or posterior cruciate ligament rupture, chondral lesions assigned to chondroplasty, meniscus rupture assigned to suturing and previous knee surgery or fracture of the affected knee, osteoarthritis, inflammatory arthritis or peripheral vascular disease (Figure 1).

### 2.2 Surgical technique and postoperative treatment

Single-incision arthroscopically assisted bone-patellar tendon-bone autograft ACLR was performed under spinal anesthesia with the patient in the supine position. A mid-third 10- or 11-mm wide patellar tendon was used as a graft. The femoral and tibial anatomic footprints with independent tibial and femoral tunnels were employed. The femoral tunnel was drilled through the antero-medial portal. The graft bone plugs were stabilized with titanium interference screws on both the femoral and tibial sides.

All subjects had undergone a standardized, moderately accelerated, 6-month rehabilitation program. A postoperative physical therapy began immediately after ACLR. Immediately, an active full extension and a flexion up to 90° of the operated knee were introduced. A free range of motion was permitted 2 weeks after the operation. Full weight-bearing (if tolerated) was allowed 1 day postoperatively. Crutches were used when needed for up to 4 weeks. No knee braces were used. Subjects were taught home exercises before discharge. Closed-chain exercises were started gradually. Running was permitted 3 months and contact sports 6–9 months postoperatively, provided that the subject had regained functional stability, muscle strength and coordination.

### 2.3 Patient-reported outcome

Knee injury and Osteoarthritis Outcome Score (KOOS) is a 42-item self-administered knee-specific Patient-relevant outcome (PRO) measure [12], which has already been adapted and validated for Polish ACL-reconstructed patients [13]. The KOOS questionnaire contains the following five subscales: pain, other symptoms, activities of daily living (ADL), sports and recreation and quality of life (QOL). A separate score ranging from 0 to 100, where 100 represents the best result, was calculated for each subscale. Participants were asked to complete the KOOS questionnaire prior to operation (assessment A) and at follow-up of a minimum of 1 year (assessment B). Those subjects who missed assessment B were sent the questionnaire by mail together with an explanatory letter. No further action was taken to include non-responders.

### 2.4 Clinical assessment

Clinical assessment was performed preoperatively (assessment A) and at 1-year follow-up (assessment B). Subjects underwent clinical evaluation, including the range of motion in the operated knee as well as manual assessment of joint stability with Lachman (according to Gurtler et al. [14]) and pivot shift test. We also reported postoperative wound healing complications, infections, the number of aspirations for hemarthrosis and the occurrence of limb swelling.

### 2.5 Primary outcome

The primary outcome was defined as the change between assessment A and assessment B in the average score for four of the five KOOS subscales, covering pain, symptoms, difficulty in sports and recreational activities, and quality of life (KOOS_4_), with scores ranging from 0 (worst) to 100 (best) [10].

Those subjects who improved in the KOOS_4_ average over time were regarded as improvers, and those who did not were called as non-improvers.

### 2.6 Assessment of activity level

Tegner Activity Scale (TAS) is a 10-level activity score reflecting the subject’s current the highest level of sports activity or other routine activities. A level of 10 represents the highest professional performance corresponding to an activity level of an elite soccer player [15,16]. The assessment was made preoperatively (assessment A) and concerned the subjects’ pre-injury activity.

### 2.7 Proposed recruitment criteria for ACLR

We retrospectively reviewed all records and proposed new recruitment criteria for ACLR. We considered subjects with ACL injury as eligible for the operation if they fulfilled the following criteria: (1) pre-injury activity level ≥7 according to the TAS, depending on the sports discipline performed, and a desire to return to professional sports activity or (2) residual knee instability following injury, defined as subjective “giving-way,” despite the 6-week structured exercise program or (3) age under 20 years at the time of operation.

### 2.8 Statistical analysis

Continuous outcomes are given as mean (standard deviation, SD) values. No prior sample size determination was made due to the observational character of the study.

A confidence interval excluding differences greater than 10 units between groups was interpreted as indicating the absence of a clinically significant difference [17].

Binary data in 2 × 2 tables were evaluated by Pearson’s chi-square test.

Analyses were performed with the use of IBM SPSS Statistics for Windows V. 24.0.0 (IBM Corp. Armonk, New York, USA). We considered a *P*-value of 0.05 or less significant. All tests were two-sided.


**Ethical approval:** The study was conducted in accordance with the ethical standards of the institutional and/or national research committee and with the 1964 Declaration of Helsinki and its later amendments or comparable ethical standards. Participation was voluntary, and withdrawal was possible at any time. The study was approved by the ethical review board at the Medical University of Łódź (approval no. RNN/804/13/KB). The patients were informed in writing and orally by the study personnel, and written informed consent was obtained from all subjects.

## 3 Results

### 3.1 Characteristics of subjects

Out of the original 320 subjects who underwent ACLR, 134 were ineligible for inclusion (Figure 1). Of 186 subjects who participated in assessment A, 77 did not respond or had incomplete documentation (follow-up rate 59%). The study group consisted of 109 subjects (78 men and 31 women) with a mean age of 30 years (median 27, range 17–65 years) who were examined at assessment B (Figure 1, Table 1).

**Table 1:** Patients’ characteristics

Characteristics	All subjects	Improvers	Non-improvers
*N* (% women)	109 (28)	49 (29)	60 (28)
Follow-up time, mean (SD), years	1.6 (0.5)	1.6 (0.5)	1.6 (0.5)
Age, mean (SD), years			
At assessment A (ACLR)	28.3 (9.4)	27.6 (9.4)	28.8 (9.5)
At assessment B	29.8 (9.4)	29.2 (9.4)	30.4 (9.4)
Body mass index, mean (SD), kg/m^2^	25.8 (4.4)	25.8 (4.6)	25.9 (4.3)

SD, standard deviation; ACLR, anterior cruciate ligament reconstruction; BMI, body mass index.

There were no statistically significant differences in age between men (mean 29, SD 8) and women (mean 32, SD 12). The mean follow-up time was 1.6 years (range 0.9 to 3.4 years) (Table 1).

To evaluate a possible inclusion bias, both the subjects who participated in assessment B and those who did not were analyzed with regard to age, gender, and BMI. No significant differences in these characteristics were found (data not shown).

### 3.2 Clinical assessment

At assessment B, a normal range of motion (0 to at least 135 degrees) was found in all patients. No patients revealed any knee instability. No postoperative complications were reported in the study group.

### 3.3 Primary outcome

The mean score of KOOS_4_ at assessment B was significantly higher than that in assessment A (71, SD 18 vs 63, SD 18, *P* < 0.001). There were 49 subjects (45%) who improved and 60 (55%) who did not improve between assessments A and B.

An analysis of respective KOOS subscales showed that the mean score difference between assessments A and B was greatest in the subscale Sports and Recreation (∆ = 13.1, *P* < 0.001) and in the KOOS subscale QOL (∆ = 11.1, *P* < 0.001). We performed a separate analysis of all patients according to the fulfillment of the proposed recruitment criteria for ACLR. The criteria were met by 58 (53%) and not met by 51 subjects (47%). An outcome improvement in KOOS_4_ at follow-up was more distinct in the group that fulfilled the recruitment criteria (Table 2).

**Table 2:** Crosstabulation of the subjects (*n*) according to fulfillment of recruitment criteria and improvement in KOOS_4_. The component bar chart below shows the same data as percentage

KOOS_4_			
Recruitment criteria	Improvers	Non-improvers	Total
Fulfilled	37	21	58
Not fulfilled	12	39	51
Total	49	60	109
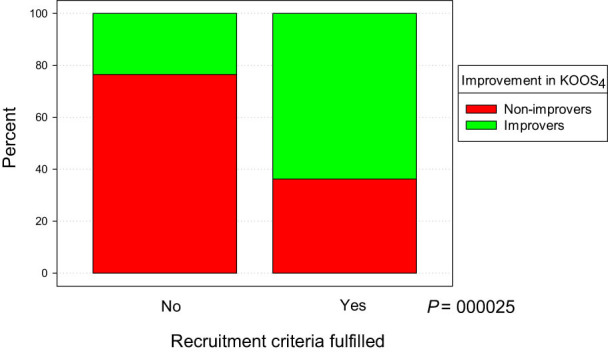			

KOOS_4_ – average score for four of the five Knee injury and Osteoarthritis Outcome Score (KOOS) subscales: pain, symptoms, sports/recreation and quality of life.

A test of the proportions for improvers versus non-improvers is shown to be 63.8 and 36.2%, respectively. A chi-square test gives a *P* < 0.0005. Expressed as an odds ratio, the OR for improvement is 5.7 times higher in the group that fulfilled the recruitment criteria than the corresponding odds in the group that did not (95% confidence interval (CI) 2.5–13.3). Further analysis revealed that the improvers scored lower in the KOOS subscale QOL at assessment A (*P* = 0.024). All other baseline KOOS scores, as well as age and BMI values, were not significantly different between improvers and non-improvers.

### 3.4 Assessment of activity level

For the entire group, a median TAS score of 4 (range 2–9) was found. A majority of the subjects assessed performed only recreational sports. Seventy-eight subjects reported TAS to be at the level of 3 or 4, and 22 subjects reported the level of 5 to 6. The group included eight semi-professional or professional competitive athletes (TAS >7 points). One person declared TAS to be at the level of 2 (data not presented).

## 4 Discussion

The study made a retrospective review of prospectively collected data from 109 subjects who had underwent ACLR. We found that the subjects who met the proposed criteria for ACLR were nearly three times more likely to improve 1 year after the operation.

As there had been no proven and well-accepted indication or recruitment criteria for ACLR before, such particular evaluation was made, to the best of our knowledge, for the first time.

The debate concerning the choice of treatment and timing of surgery is ongoing [18]. The decision regarding the choice of operative or non-operative treatment is usually made by the responsible orthopedic surgeon in communication with the patient and the treating physical therapist. The operation is usually performed in subjects with dynamic knee instability who intend to participate in level-I sports [4]. The patient’s young age (if skeletally mature) and his or her preference for surgery [5] may also prompt the decision to perform ACLR. However, in patients with tears in the proximal part of ACL, an early intervention with primary ACL repair (with or without additional internal bracing) in the days to weeks after injury is now postulated [19]. Some authors suggest that more rapid restoration of tibiofemoral stability results in less knee joint awareness [20], reduces the risk of chondral and meniscal damage [21] and consequently further osteoarthritis [22].

In a previous study, we suggested that subjects assigned to ACLR (1) should have a residual dynamic knee instability following the knee injury despite the 6-week structured exercise program or (2) were at TAS pre-injury level ≥7, depending on the sports discipline performed and (3) should want to return to a professional sports activity [23]. For skeletally mature adolescents, TAS ≥4 and residual dynamic knee instability were accepted as an indication for surgery [23]. We found that these criteria were met by only 44% of subjects assessed and that over one-fourth of all subjects underwent ACLR despite that they demonstrated superior stability of the knee joint and had successful preoperative outcomes in the KOOS subscales [23]. The study group assessed in our present report is based on the same cohort (but supplemented with subjects operated on later). Since our modified recruitment criteria were fulfilled by only 53%, nearly half of the subjects examined should not have undergone ACLR.

One of the criteria that we set for performing ACLR was a wish to return to sport. As has already been observed, that motivation was a key issue in ACL rehabilitation [24,25,26] and, consequently, achieving a successful outcome. Approximately 90% of patients with ACL tears seek surgical reconstruction and often want to return to their pre-injury level of function as soon as possible postoperatively [27]. Athletes who do not desire to return to their earlier activity level do not have an early indication for surgery.

Measuring the outcome of ACLR is challenging per se. The absence of a symptomatic knee joint instability, quadriceps and hamstrings strength symmetry and, above all, return to sports have traditionally been the most referenced measures of success [28]. A return to pre-injury activity was chosen as the primary outcome in several studies [8,29,30,31]. However, most of these reports used non-validated, self-report measures of return to sport [8,29,30].

Since we did not have clinical data concerning knee function and complaints from before the injury, and along with the current recommendations to preferably assess patients’ satisfaction, we decided to assume that improvement could be defined as a change in the mean score of the KOOS subscales between pre-surgery assessment and follow-up. Although the KOOS subscale ADL has previously been reported as not sensitive enough to demonstrate clinical changes in patients undergoing ACLR, Frobell et al. suggested that the analysis could be limited to the remaining four KOOS domains [10,11]. Such an approach was again used in a recent report on results of arthroscopic meniscectomy and exercise therapy in patients with degenerative meniscus tears [32]. Drawing on these findings, we chose to use KOOS_4_ as the primary outcome although we acknowledge that the ADL subscale is also responsive.

Our method, albeit subjective, is based on a validated measurement tool [13], and thus is reliable and reproducible. A similar approach was used by Dunn et al. [3], Spindler et al. [33], and Chen et al. [34] who defined improvement as a change in the mean score exceeding the clinically meaningful effect. A threshold of change regarded to be meaningful was set at 11 points for the International Knee Documentation Committee (IKDC) Subjective Knee Form and 8 points for the KOOS. The latter was lower than in our study, however within the previously recognized limits [17]. In other studies, a successful outcome following management of torn ACL was defined either as a score greater than the subject-specific age- and gender-matched population means (using the IKDC) [35] or 95% CI (using the KOOS) [36,37]. This corresponds to a score exceeding the threshold between 85% and 90% of the maximum value for respective functional PROs [28].

It has already been identified that potential predictors of a successful outcome following ACLR included younger age, “normal” knee flexion and extension strength, and no previous knee surgery [38,39,40,41], whereas factors such as smoking, preoperative quadriceps strength deficits, limitations of a range of motion, knee laxity and concomitant meniscus injury were identified as predictors of poor postoperative results and/or ACLR failure (Table 3) [38,39,41,42,43,44]. However, knowledge of these factors has not yet resulted in establishing any criteria to select subjects who would benefit from ACLR. The present study undertakes such an attempt. We observed that the number of subjects who improved was significantly higher among those who fulfilled the criteria for ACLR than those who did not, but still not larger than 64%. However, even if we set the recruitment criteria more rigorously, and consequently had fewer subjects who met them, we could expect that there would still be some individuals who would not improve. The outcome of ACLR presumably depends on other factors, not investigated in this study or even not measurable with the use of PROM.

**Table 3:** Selection of studies that investigated the prediction of outcome following ACLR

Study	Journal	Year	Level	Study design/method	Outcome measures	Follow-up	Number of enrolled participants/number of studies	Results
Robb et al. [44]	*Knee Surg Sports Traumatol Arthrosc*	2015	2	Prospective cross-sectional	IKDC, KOOS, Lysholm score, TAS, clinical tests and performance tests	12 months	94 subjects	Meniscus deficiency predicts poor outcome
Parkinson et al. [42]	*Am J Sports Med*	2017	3	Case-control	IKDC, KOOS, Lysholm score, TAS, clinical tests	>24 months	123 subjects	Meniscus deficiency predicts poor outcome
Scherer et al. [40]	*The Knee*	2015	3	Retrospective cross-sectional	Lysholm score, KOOS	6 months	118 subjects	Age ≤30 years, higher subjective knee score, normal quadriceps and hamstring strength, no previous knee surgery predict rapid recovery following single-bundle ACLR
Paterno et al. [43]	*Orthop J Sport Med*	2017	3	Case-control	IKDC, KOOS, functional performance tests	4.5 months at average	114 subjects	Age <19 years, higher knee-related confidence, performance tests are predictive for second ACL injury after ACLR
Chen et al. [34]	*Orthop J Sport Med*	2018	3	Retrospective cross-sectional	PROMIS	24 months	233 subjects	Preoperative PROMIS scores predict both successful and poor postoperative outcome
De Valk et al. [38]	*Arthroscopy*	2013	3	Meta-analysis	TAS, clinical tests	>12 months	18 studies (1 RCT, 8 prospective, 9 retrospective)	Age ≤30 years, male sex, early reconstruction predict successful outcome
								Smoking, BMI >30, quadriceps strength deficits, and range-of motion deficits predict poor outcome
Van Melick et al. [41]	*Br J Sports Med*	2016	3	Meta-analysis	TAS, clinical tests, BMI	>12 months	10 studies (1 systematic review, 1 RCT, 8 prospective)	Prehabilitation, age <30 years, higher preoperative TAS scores, early reconstruction predict higher activity 2 years after ACLR
								Deficit of extension and quadriceps strength predicts poor outcome
Hamrin Senorski et al. [39]	*Br J Sports Med*	2019	3	Meta-analysis	KOOS, EQ-5D, TAS	—	35 studies based on the Swedish, Danish and Norwegian registries	Age ≤30 years, male sex, non-smoking, hamstring tendon autograft, and no concomitant knee injuries predict successful outcome

ACL, anterior cruciate ligament; ACLR, anterior cruciate ligament reconstruction; BMI, body mass index; EQ-5D, EuroQol 5-Dimension Questionnaire; IKDC, International Knee Documentation Committee score; KOOS, Knee injury and Osteoarthritis Outcome Score; PROMIS, Patient-Reported Outcomes Measurement Information System; RCT, randomized clinical trial; TAS, Tegner Activity Scale.

The retrospective approach and the limited sample of patients (59% follow-up rate) are in fact the most important limitations of our study. Since the loss to follow-up rarely occurs randomly, the follow-up rate under the recommended threshold of 60% can be associated with a potential bias [45]. The sample is also too small to provide a more detailed analysis of factors such as concomitant meniscus injury. Another important weakness of the present analysis is that the follow-up period is much shorter than 10 years recommended for analyses with PRO scores [46]. However, it has been shown that patients undergoing ACLR assessed with the KOOS reported subjective maximal improvement 12 months postoperatively and exhibited no additional significant improvement beyond that point [47]. Return to sports also happens usually within the first year after ACLR [48,49]. Since the follow-up time in our study group varied relatively much, from 0.9 to 3.4 years, some subjects might have had more time to change their functional status. Thus, the results of the study should be interpreted with caution.

Strengths of this study include the facts that assessments were carried out in subjects who constituted a typical and well-defined group with characteristics corresponding to the cohorts described in several registries [50], and with the use of validated outcome measures. All patients were operated by the same surgeon using the same method (DW) and followed up by investigators who were not directly involved in the initial management of the patients (MW, AS), which reduced a potential bias.

## 5 Conclusion

Our results show that fulfillment of recruitment criteria was a strong predictive factor for clinical improvement in short- to medium-term follow-up after ACLR.

Further research in a larger study group is however needed to confirm our preliminary observations and thus establish reliable recruitment criteria in order to avoid overtreatment in ACL-deficient subjects.

## Abbreviations

ACL: anterior cruciate ligament
ACLR: anterior cruciate ligament reconstruction
ADL: activities of daily living
CI: confidence interval
IKDC: International Knee Documentation Committee
KOOS: Knee Injury and Osteoarthritis Outcome Score
PRO: patient-reported outcome
SD: standard deviation
TAS: Tegner activity score
QOL: quality of life
